# Rice Water-Fried Atractylodis Rhizoma Relieves Spleen Deficiency Diarrhea by Regulating the Intestinal Microbiome

**DOI:** 10.1155/2023/1983616

**Published:** 2023-02-07

**Authors:** Chunping Xiao, Meiyi Wang, Xueqing Yang, Jin Sun, Lili Weng, Zhidong Qiu

**Affiliations:** School of Pharmaceutical Sciences, Changchun University of Chinese Medicine, Changchun, China

## Abstract

**Background:**

Spleen deficiency diarrhea (SDD) is a common Traditional Chinese Medicine (TCM) gastrointestinal condition, the causes of which include dysfunction of the intestinal barrier and microbiota. Rice water-fried Atractylodis Rhizoma (RAR) is a commonly used drug to treat this condition, but its mechanism remains unclear. This study explored the related mechanisms of ethanolic extract of rice water-fried Atractylodis Rhizoma (EAR) in the treatment of SDD by examining changes in the intestinal microbiota.

**Method:**

Wistar rats were randomly divided into 4 groups including the control, model, EAR low, and high-dose groups, 6 rats in each group. All rats, except the control group, were induced to develop SDD by a bitter-cold purgation method with rhubarb. The therapeutic effect of EAR on SDD was evaluated by pathological sections, inflammatory indicators (TNF-*α*, IL-1*β*, and IL-10), gastrointestinal-related indicators (GAS, DAO, D-lactate, VIP, and SIgA), and intestinal flora (bacteria and fungi) analysis.

**Results:**

The results showed that the developed SDD rat model (model group) showed weight loss, decreased food intake, and increased fecal moisture content. Compared with those of the control group, the levels of TNF-*α*, IL-1*β*, DAO, D-lactate, and VIP in the model group were significantly increased, but the levels of IL-10, GAS and SIgA were significantly decreased (*p* < 0.05). However, the indicators were significantly improved after EAR treatment, indicating that EAR maintained the balance of pro- and anti-inflammatory cytokines and reduced gastric emptying, thereby protecting intestinal barrier function, alleviating intestinal mucosal injury, and relieving SDD by regulating the release of neurotransmitters. EAR was also shown to prevent infection by promoting the accumulation of noninflammatory immunoglobulin SIgA and improving intestinal mucosal immunity to inhibit the adhesion of bacteria, viruses, and other pathogens. Intestinal microbiome analysis showed that the intestinal bacteria and fungi of SDD model rats changed greatly compared with the control group, resulting in intestinal microecological imbalance. The reversal in the composition of the flora after EAR treatment was mainly characterized by a large enrichment of beneficial bacteria represented by *Lactobacillus* and a decrease in the abundance of potentially pathogenic fungi represented by *Aspergillus*. Thus, it was speculated that EAR primarily functions to alleviate SDD by increasing the abundance of beneficial bacteria and reducing the abundance of potentially pathogenic fungi.

**Conclusion:**

The strong therapeutic effect of EAR on SDD suggests that it is a promising treatment for this condition.

## 1. Introduction

Spleen deficiency diarrhea (SDD) is a common clinical gastrointestinal syndrome. According to Traditional Chinese medicine (TCM), the main cause of SDD is gastrointestinal conduction dysfunction caused by spleen deficiency, internal dampness, pulmonary asthenia, and stomach disharmony [1]. Spleen deficiency is not conducive to transport and a weak stomach fails to digest processed food, resulting in water and food retention and lower spleen yang, which eventually leads to diarrhea [2]. The main characteristics of SDD are loose stools, accompanied by abdominal pain and muscle fatigue [3]. Treatment is focused on increasing vital energy and invigorating the spleen. Atractylodis Rhizoma has the effect of drying dampness and invigorating spleen, but raw Atractylodis Rhizoma has strong dryness and needs to be processed to relieve dryness. Rice water stir-frying is a major feature of Atractylodis Rhizoma processing. It was recorded as early as in the “Lei-Gong-Pao-Zhi-Lun” in the Southern and Northern Dynasties of China. Ming Dynasty “Ben-Cao-Gang-Mu” also recorded rice water taste sweet and cool, with heat, cool blood, and detoxification effect. Rice water-fried Atractylodis Rhizoma (RAR) is considered to have the effect of relieving dryness and invigorating spleen and is especially good at invigorating spleen compared with other processed products. Therefore, RAR is often used to treat SDD [4]. However, unlike single-component chemicals, this and other herbal medicines are often used to treat patients holistically [1]. System biology, the exploration of complex interactions within biological systems components, has made it possible to study specific disease symptoms along with the mechanism of action of different TCM herbs [5]. However, due to the diversity of TCM components and the complexity of drug-human interactions, there is very little understanding of the pharmacodynamics and mechanisms of action of specific herbal medicines [6]. This has greatly limited their development.

Recent studies have suggested that the intestinal microbiome plays a role in SDD progression [1] and that several TCM components interact directly with intestinal bacteria and fungi [7, 8]. By specifically regulating the composition and diversity of intestinal microflora, TCM (1) improves intestinal inflammation and relieves pathological damage [9], (2) changes the bioavailability and bioactivity/toxicity of specific TCM components [10], and (3) allows synergy or antagonism to occur between particular TCM chemicals [11]. Studies indicate that Atractylodis Rhizoma treatment can restore the richness and diversity of intestinal bacteria that is lost in SDD model rats. While *Helicobacter pylori* and other pathogenic bacteria decrease significantly after administration of Atractylodis Rhizoma, beneficial bacteria such as *Lactobacillus* increase [1]. Several other herbal extracts are shown to alleviate diarrhea by regulating the growth of intestinal bacteria, such as *Pueraria lobata* [12] and *Cortex Phellodendri* [8]. While intestinal fungi are also important for maintaining intestinal homeostasis [13], there has been little research on these organisms because they only account for a small proportion of the intestinal microbiome and most are uncultured [7]. The intestinal fungal biome has attracted more attention in recent years as a result of its potential correlation with various digestive diseases [14]. Intestinal fungal flora are shown to be involved in low-grade inflammation, intestinal ecological disorders, and brain-gut interactions [13]. The pattern recognition receptors C-type lectin receptors (CLRs) and Toll-like receptors (TLRs) are able to recognize fungal components (*β*-glucan, chitin, and mannan) and initiate downstream signaling pathways that activate proinflammatory responses and low-grade intestinal inflammation [15]. Overall, a healthy gut microbiome is important for intestinal mucosal protection, immune homeostasis, and pathogen suppression [16].

The current study established an SDD model and analyzed the effects of an ethanolic extract of rice water-fried Atractylodis Rhizoma (EAR) on immune function and intestinal flora (bacteria and fungi) in SDD rats. The study sought to clarify the correlation between RAR and the intestinal microbiome; to describe interactions among intestinal microorganisms, drugs, and organisms; and to provide a theoretical reference for expanding the clinical application of RAR.

## 2. Materials and Methods

### 2.1. Materials and Chemicals

The RAR used in this study was Reference 2020 edition Jilin Province Chinese herbal pieces processing standard laboratory-made [17]. Rhubarb (*Rheum officinale* Baill.) was purchased from Anxing Chinese Medicine Pieces Co., Ltd. (Anguo, China). Tumor Necrosis Factor Alpha (TNF-*α*), Interleukin 1 Beta (IL-1*β*), Interleukin 10 (IL-10), Gastrin (GAS), Diamine Oxidase (DAO), D-lactate, Vasoactive Intestinal Peptide (VIP), and Secreted Immunoglobulin (SIgA) ELISA kits were purchased from Jiangsu Enzyme Immunoglobulin Co., Ltd. (Yancheng, China).

### 2.2. Experimental Animals

Specific Pathogen Free (SPF) Wistar rats weighing ~200–220 g were purchased from Liaoning Changsheng Biotechnology Co., Ltd. (Liaoning, China). (Note: due to the smaller spleen of germ-free rats compared to normal rats, it is not conducive to the study of SDD. Therefore, we chose to exclude Yersinia enterocolitica, dermatopathogenic fungi, and Klebsiella pneumoniae, and other pathogens SPF rats were studied.) The rats were raised in a SPF animal room with an independent ventilation system and standard temperature and humidity. All rats were fed using a standard 12–12 h light-dark cycle and allowed to adapt for 1 week prior to the study. The rats were provided with a standard diet (calorie composition: 65.5% carbohydrate, 10.3% fat, and 24.2% protein) and water. The experimental design was strictly in accordance with the principles and experimental requirements of the Animal Ethics Committee of Changchun University of Chinese Medicine (No. 2020317).

### 2.3. Reagent Preparation

#### 2.3.1. Rhubarb Aqueous Extract Preparation

Chopped rhubarb (100 g) was decocted with 500 ml water for 30 min. After filtering, the dregs were decocted in 300 ml of water for 20 minutes. Combine the two filtrates, and transfer to a beaker. The filtrate was dried at 60°C under reduced pressure, concentrated to 50 ml to obtain a rhubarb decoction (containing 2 g·ml^−1^ raw drug), and stored at 4°C for future use.

#### 2.3.2. EAR Preparation

The appropriate amount of RAR was crushed 10x using a 50 mesh sieve with 80% ethanol solution and soaked overnight. The following day, the RAR was ultrasonic extracted 3 times for 2 h each and combined with the filtrate. The alcohol was recovered, and the RAR was concentrated to 756 mg·ml^−1^ for the high-dose group. A portion was diluted to 189 mg·ml^−1^ for the low-dose group.

### 2.4. GC-MS Analysis of EAR

The composition of EAR was analyzed by gas chromatography-mass spectrometry (GC-MS, Trace 1310 gas chromatograph and TSQ 8000 mass spectrometer) system (Thermo, USA). Chromatographic separation was achieved using a Rtx-5 column (30 m × 0.32 mm id × 0.25 *μ*m film thickness) purchased from Thermo (USA). The carrier gas was high purity He (99.999%, flow rate: 1 ml·min^−1^), the injection volume was 1 *μ*l, and the inlet temperature was 250°C. The initial column temperature was 60°C, and the temperature was increased to 280°C at 15°C·min^−1^ and maintained for 5 min. The mass spectrum was bombarded using an EI ion source with an ionisation energy of 70 eV, an ion source temperature of 250°C and an interface temperature of 250°C, and a scan mass range of 30 to 500 amu. Solvent excision time is 4 min, start time is 4.0 min, and end time is 20.0 min.

### 2.5. SDD Model and Group Treatments

**Figure 1:**
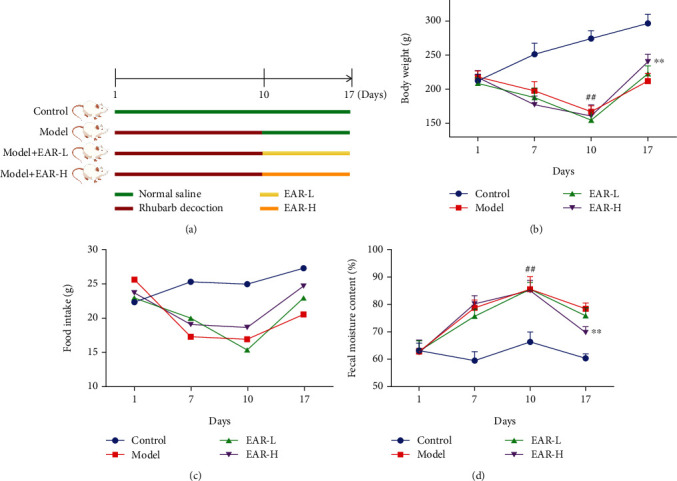
Effect of EAR on the body weight, food intake, and fecal moisture content of SDD rats. Note: compared with the control group: ^##^*p* < 0.01; compared with the model group: ^∗∗^*p* < 0.01. Data are expressed as the mean ± SD; *n* = 6.

SDD rats were prepared as described by Shi et al. with slight modifications [1]. Specifically, 24 male Wistar rats were randomly divided into four groups, a control group, a model group, an EAR-L group, and an EAR-H group with six rats per group. The rats in each group except the control group were administered 20 ml·kg^−1^ rhubarb decoction intragastrically twice a day in the morning and evening for ten days. The SDD model was evaluated and considered successful based on the general behavior of the rats and the moisture content of their feces. Subsequently, intragastric administration was performed at 10 ml·kg^−1^. The low-dose treatment group was administered 189 mg·ml^−1^ EAR while the high-dose group was administered 756 mg·ml^−1^ EAR. The control and model groups were given normal saline gavage once a day for 7 days. The treatments are described in Figure 1(a).

### 2.6. Sample Collection

On days 0, 7, 10, and 17 after treatment, fresh feces were collected from rats before intragastric administration and dried at 105°C for 5 h to determine the moisture content of feces. At 12 h after the last administration, fresh feces (3–5 pellets per rat) were collected from three rats in each group using abdominal massage under aseptic conditions. The pellets were stored in sterile tubes at -80°C for analysis of the intestinal flora. The rats were then anesthetized with 20% urethane (0.8 ml·100 g), and blood was collected from the abdominal aorta. The blood was centrifuged at 3,000 rpm at 4°C for 20 min. After separation of the serum, TNF-*α*, IL-1*β*, IL-10, GAS, DAO, D-lactate, VIP, and SIgA levels in the rat sera were measured by enzyme-linked immunosorbent assay (ELISA). Finally, a ~4 cm section of the rat colon was cut, fixed with 4% paraformaldehyde, embedded in paraffin, sliced into 4 *μ*m sections, and hematoxylin-eosin (HE) stained. The remaining colons were stored at -80°C, and TNF-*α*, IL-1*β*, and IL-10 levels in the colon tissues were measured by ELISA. The colon tissue sample was weighed 0.1 g, added to 0.9 ml precooled 0.01 mol·l^−1^ phosphate-buffered saline (PBS), and ground into a homogenate with liquid nitrogen. The slurry was centrifuged at 5,000 r·min^−1^ at 4°C for 15 min to remove the cell debris; then, the supernatant was then taken for determination.

### 2.7. Analysis of the Intestinal Microbiome

DNA was extracted from the feces of the four treatment groups using a Power Soil DNA Isolation Kit (Macherey-Nagel, Duren, Germany). DNA concentration and purity were assessed by 1.8% agarose gel electrophoresis and NanoDrop One. Intestinal bacteria were isolated using a 16S full-length PCR amplification system with the following primers: 27F (5′-AGR GTT TGA TYN TGG CTC AG-3′) and 1492R (5′-TASGGHTACCTTGTTASGACTT-3′). The reaction mixture included 50 ng template DNA, 3.5 *μ*l NFW, 5 *μ*l KOD ONE MM, 0.5 *μ*l 10 *μ*M forward primer, and 0.5 *μ*l 10 *μ*M reverse primer, and ddH_2_O was added for a final volume of 10 *μ*l. The reaction procedure included a 95°C predenaturation for 2 min, 25 cycles of 98°C for 10 s, 55°C for 30 s, and 72°C for 90 s, and a final extension at 72°C for 2 min.

The intestinal fungal ITS full-length PCR amplification system included the following primers: ITS1F (5′-CTT GGT TTA GAG GAA GTA A-3′) and ITS4 (5′-TCC TCC GCT TAT TGA TAT GC-3′). The reaction mixture included 50 ng template DNA, 3.9 *μ*l NFW, 5 *μ*l KOD ONE MM, 0.3 *μ*l 10 *μ*M forward primer, and 0.3 *μ*l 10 *μ*M reverse primer, and ddH2O was added for a final volume of 10 *μ*l. The reaction procedure included a 95°C predenaturation for 5 min, 8 cycles of 95°C for 30 s, 55°C for 30 s, and 72°C for 45 s, 24 cycles of 95°C for 30 s, 60°C for 30 s, and 72°C for 45 s and a final extension at 72°C for 5 min. PCR products were examined for integrity by electrophoresis, quantified by NanoDrop One, mixed at a mass ratio of 1 : 1, and recovered using 0.8X Magic Pure Size Selection DNA Beads. A database was constructed according to the NEB Next Ultra TM DNA Library Prep Kit for Illumina standard process, and the high-throughput sequencing platform, PacBio Sequel, was used for sequencing. The original off-camera subreads were corrected to obtain Circular Consensus Sequencing (CCS) sequences (SMRT Link, version 8.0), and Lima (version 1.7.0) software was used to identify the CCS sequences of different samples using barcode sequences. Chimeras (UCHIME, version 8.1) were removed to obtain high-quality CCS sequences. The sequencing work was completed by BioMarker Technologies Co., Ltd. (Beijing, China). All sequence data were submitted to the Sequence Read Archive (bacterial accession number: PRJNA831020; fungal accession number: PRJNA831033) and are freely available at the NCBI (https://www.ncbi.nlm.nih.gov/sra/PRJNA831020;https://www.ncbi.nlm.nih.gov/sra/PRJNA831033).

### 2.8. Sequence Data Processing and Statistical Analysis

Cluster analysis was performed using USEARCH (version 10.0) software at a 97% similarity level. Species abundance and community composition at the phylum and genus levels were analyzed using MEGAN5 (http://ab.inf.uni-tuebingen.de/software/megan5/). Mothur (versionv.1.30) (http://www.mothur.org) software was used to determine the richness and diversity indexes of the intestinal microbiota of rats in each treatment group. The distance matrix between samples was obtained based on the weighted-Unifrac distance algorithm, and a sample clustering tree and abundance histogram were constructed using the unweighted group average (UPGMA) method. The Metastats (http://metastats.cbcb.umd.edu/) software was used to run a *t*-test to compare intestinal microbiota species abundance of the different groups. Principal component analysis (PCA) was conducted by WGCNA, stat, and ggplot2 packages in R software (Version 3.6.0). Significant *p* values in the LEfSe analysis were calculated using the Wilcoxon rank-sum test, and the LDA was set to 4.0. SPSS 21.0 and GraphPad prism 8.0 software were used to analyze and plot differences in the related indexes of rats in the different treatment groups. All values were represented as the mean ± standard deviation (SD). Statistical comparisons were performed using a one-way analysis of variance (ANOVA) with Students' *t*-test, and *p* < 0.05 was considered statistically significant.

## 3. Results

### 3.1. Identification of Compounds in the GC-MS Chromatogram

GC-MS was used to identify the chemical composition of EAR (Supplementary Figure 1). The 10 chemical components that were present in the highest concentrations were separated and identified (Table 1).

### 3.2. Effect of EAR on the Body Weight, Food Intake, and Fecal Water Content of SDD Rats

After ten days of rhubarb modeling, rats in the model and treatment groups had greater weight loss (Figure 1(b)), greater loss of appetite (Figure 1(c)), and higher fecal moisture content (Figure 1(d)) than that those the control group (*p* < 0.01), indicating that the SDD model was successfully established. After 7 days of treatment, body weight and food intake were notably higher for rats in the EAR-H group than the model group while fecal water content was significantly lower (*p* < 0.01), indicating that high-dose EAR could effectively alleviate SDD symptoms in rats.

### 3.3. Effect of EAR on Inflammatory Cytokine Production and Colonic Pathology in SDD Rats

**Figure 2:**
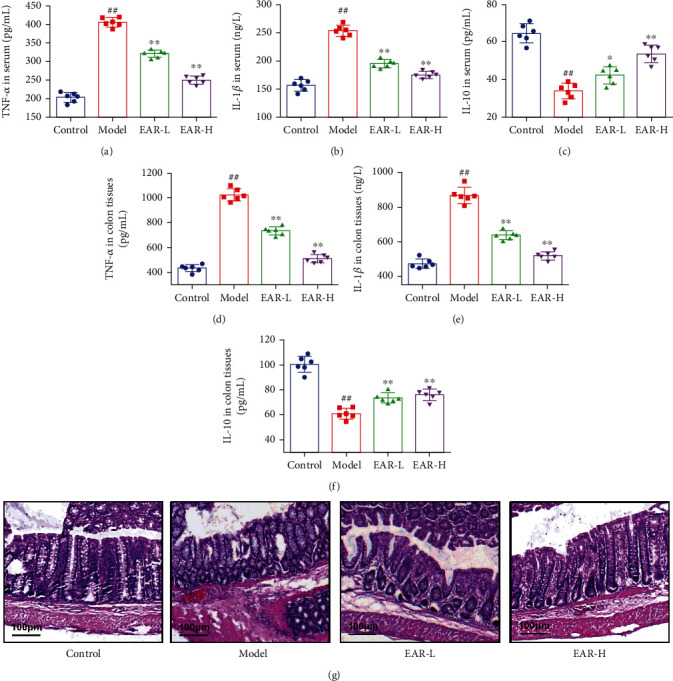
Effect of EAR on colonic inflammation and pathology in SDD rats. Note: compared with the control group: ^##^*p* < 0.01; compared with the model group: ^∗^*p* < 0.05 and^∗∗^*p* < 0.01. Data are expressed as the mean ± SD; *n* = 6.

Levels of the proinflammatory cytokines, TNF-*α* and IL-1*β*, were significantly higher in the serum and colonic tissue of the model group than in the control group, while levels of the anti-inflammatory cytokine, IL-10, were significantly lower (*p* < 0.01), indicating that the model rats produced an inflammatory response (Figures 2(a)–2(f)). Compared with the model group, TNF-*α* and IL-1*β* were significantly lower, while IL-10 was significantly higher in the treatment group (*p* < 0.01), and the EAR-H group showed a prominent regulatory effect. Compared with the control group, hematoxylin-eosin staining showed obvious pathological changes (few goblet cells, high inflammatory cell infiltration, and crypt damage) in the colonic tissue of rats in the model group (Figure 2(g)). While mice in the EAR-L group had some epithelial cell damage and inflammatory cell infiltration, mice in the EAR-H group had no obvious damage to the intestinal structure, uniform arrangement of goblet cells, and reduced infiltration of inflammatory cells.

### 3.4. Effect of EAR on Serum Gastrointestinal-Related Indexes in SDD Rats

**Figure 3:**
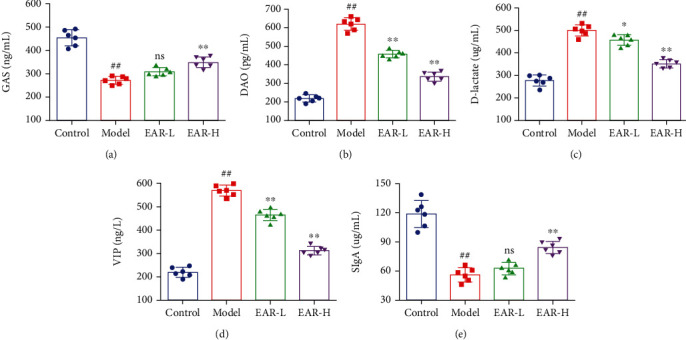
Changes in gastrointestinal-related indexes in the serum of SDD rats. Note: compared with the control group: ^##^*p* < 0.01; compared with the model group: ^∗^*p* < 0.05 and^∗∗^*p* < 0.01. Data are expressed as the mean ± SD; *n* = 6.

Serum gastrin (GAS) levels were 40.04% lower in the model group than in the control group (*p* < 0.01) indicating that gastric emptying function was accelerated (Figure 3(a)). After treatment, GAS levels in the EAR-H and EAR-L groups were 28.06% and 13.33% higher than in the model group (*p* < 0.05), indicating that EAR significantly reduced gastric emptying function and improved diarrhea. Serum Diamine Oxidase (DAO), D-lactate, and Vasoactive Intestinal Peptide (VIP) levels were 183.31%, 79.92%, and 158.66% higher in the model group than in the control group while SIgA was 52.61% lower (*p* < 0.01), indicating that intestinal mucosal permeability changed in the model group and mucosal barrier function became impaired (Figures 3(b)–3(e)). DAO, D-lactic acid, and VIP levels were 45.67%, 29.62%, and 45.21% lower in the EAR-H group than in the model group (*p* < 0.05), while SIgA was 49.71% higher (*p* < 0.01), indicating that EAR improved intestinal mucosal permeability, increased immune function and repaired the intestinal mucosal injury.

### 3.5. Effect of EAR on the Intestinal Microbiome *α*-Diversity of SDD Rats

**Figure 4:**
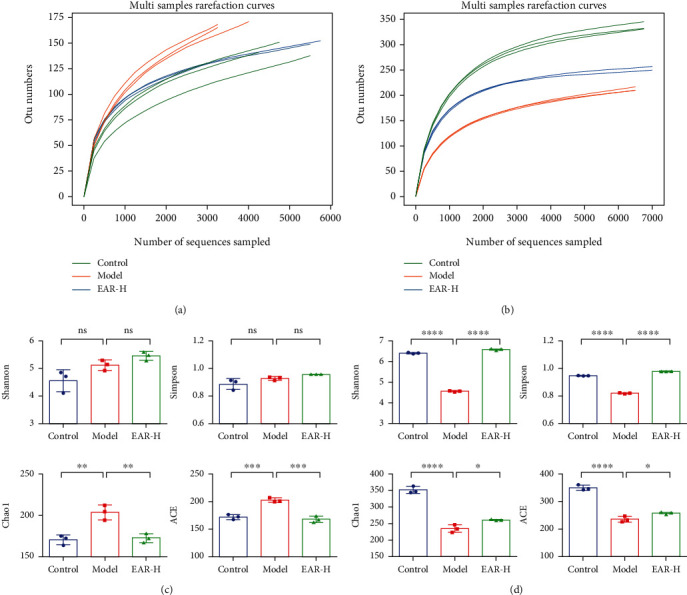
High-throughput sequencing and alpha diversity index of the intestinal microflora of SDD rats. Rarefaction curve of intestinal bacteria (a) and fungi (b); alpha-diversity of intestinal bacteria (c) and fungi (d). Note: data are expressed as the mean ± SD; ^∗^*p* < 0.05,  ^∗∗^*p* < 0.01,  ^∗∗∗^*p* < 0.001 and^∗∗∗∗^*p* < 0.0001; *n* = 3.

In view of the good effect of EAR at high doses, an in-depth assessment of the microbiome in the control, model, and EAR-H groups was assessed in subsequent experiments. Overall changes in the intestinal bacteria of rats in the different treatment groups were assessed using 16S rDNA high-throughput sequencing. A total of 49,236 raw Circular Consensus Sequencing (CCS) were obtained, of which 45,231 effective CCS were kept after removing low-quality sequences from the original data using quality detection. By clustering effective CCS at the 97.0% similarity level, 341 bacterial OTUs were identified. The different treatment groups had 53 OTUs in common, of which 104, 43, and 13 were unique to the control, model, and EAR-H groups, respectively (Supplementary Figure 2A). As the number of sampling sequences increased, the sparse curve showed that the number of OTUs in the sample reached the platform period, indicating that the sequencing work was sufficient to reflect the real conditions of the sample (Figure 4(a)). The alpha diversity analysis uses OTU clustering results to detect species richness and diversity, the Shannon and Simpson index reflects species diversity, and the Chao1 and ACE indexes reflect species richness. While the Shannon and Simpson indexes were similar between the model and control groups (*p* > 0.05), the Chao1 and ACE indexes were significantly higher in the model group (*p* < 0.05) (Figure 4(c)). Chao1 and ACE indexes decreased after EAR treatment, indicating that this medicine effectively regulated the intestinal bacterial richness of SDD rats and improved the intestinal microenvironment.

ITS high-throughput sequencing was used to explore changes in the intestinal fungi of rats. A total of 66,367 raw CCS were obtained by sequencing, of which 64,412 effective CCS were obtained by quality control filtering. Effective CCS were clustered at the 97.0% similarity level, and 652 fungal OTUs were identified. There were 197, 84, and 186 unique OTUs in the control, model, and EAR-H groups, respectively (Supplementary Figure 2B). The sparse curve showed that the number of OTUs in the sample was close to saturation, indicating that the detection sample was sufficient and the sequencing results were reliable (Figure 4(b)). The alpha diversity of each group is shown in Figure 4(d). The Shannon and Simpson indexes were significantly lower in the model group than in the control group (*p* < 0.05) and significantly higher in the EAR group than in the model group (*p* < 0.05). Trends in the Chao1 and ACE indexes matched those of the Shannon and Simpson indexes. These findings indicated that EAR plays a role in reshaping the richness and diversity of intestinal fungi.

### 3.6. Effect of EAR on the Intestinal Microbiome Composition of SDD Rats

**Figure 5:**
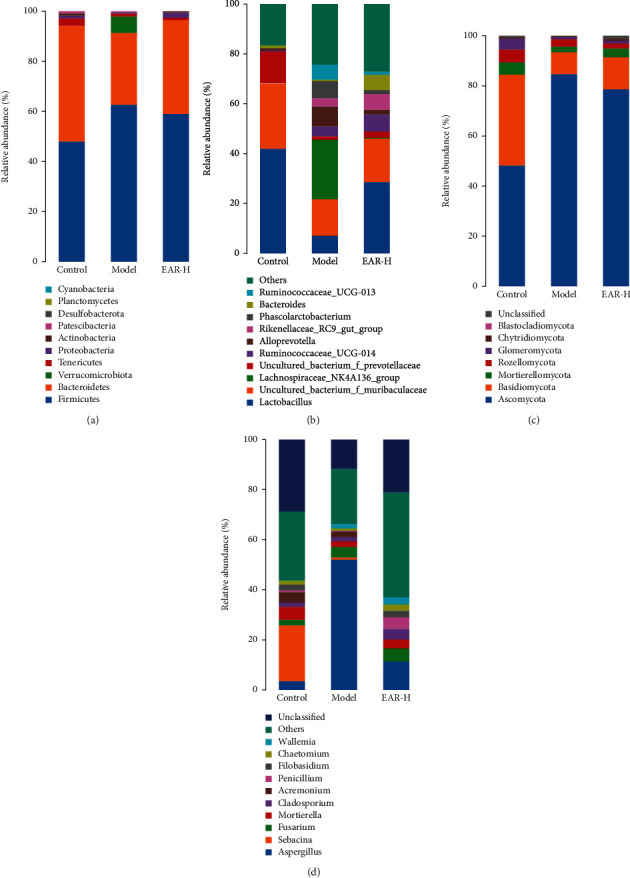
Relative abundance of intestinal bacterial communities at phylum (a) and genus (b) levels and fungal communities at phylum (c) and genus (d) levels of rats in different treatment groups.

The intestinal bacteria of rats were classified and identified based on the OTUs. At the phylum level (Figure 5(a)), Firmicutes, Bacteroidetes, and Tenericutes were the primary intestinal bacteria. The relative abundance of the ten most abundant bacterial phyla in each treatment is shown in Supplementary Table 1. The relative abundances of Firmicutes and Bacteroidetes were 47.92% and 46.33%, respectively, in the control group. The relative abundance of Firmicutes was higher while the relative abundance of Bacteroidetes was lower in the model group than in the control group. The dominant bacterial flora in the EAR group was comparable to the control group, indicating that EAR had a moderating effect on intestinal bacterial dysfunction in SDD rats. At the genus level (Figure 5(b)), the top ten dominant bacterial genera were *Lactobacillus*, *uncultured_bacterium_f_Muribaculaceae*, *Lachnospiraceae_NK4A136_group*, *uncultured_bacterium_f_Prevotellaceae*, *Ruminococcaceae_UCG-014*, *Alloprevotella*, *Rikenellaceae_RC9_gut_group*, *Phascolarctobacterium*, *Bacteroides*, and *Ruminococcaceae_UCG-013*, and their relative abundance is listed in Supplementary Table 2. The relative abundances of *Lactobacillus*, *uncultured _ bacterium_f_Muribaculaceae*, and *uncultured_bacterium_f_Prevotellaceae* were lower in the model group than in the control group while the relative abundances of *Lachnospiraceae_NK4A136_group*, *Ruminococcaceae_UCG-014*, *Alloprevotella*, *Rikenellaceae_RC9_gut_group*, *Phascolarctobacterium*, and *Ruminococcaceae_UCG-013* were higher. The relative abundance of *Lactobacillus*, *uncultured_bacterium_f_Muribaculaceae*, *Lachnospiraceae_NK4A136_group*, and *uncultured_bacterium_f_Prevotellaceae* recovered to normal levels in the EAR group. Of these, the relative abundance of *Lactobacillus*, the primary beneficial bacteria, was 75.46% higher in the EAR group than in the model group, while the relative abundance of *Lachnospiraceae_NK4A136_group* and *Alloprevotella*, potentially harmful intestinal bacteria, were 98.77% and 77.53% lower in the EAR group than in the model group, respectively.

The intestinal fungi of rats were classified and identified based on OTUs. At the phylum level (Figure 5(c)), Ascomycota, Basidiomycota, Mortierellomycota, Rozellomycota, and Glomeromycota were the primary intestinal fungi. The relative abundance of the seven most abundant fungal phyla in each treatment is shown in Supplementary Table 3. Of these, the relative abundances of Ascomycota and Basidiomycota were 48.17% and 36.34%, respectively, in the control group. The relative abundance of Ascomycota was higher in the model group than in the control group, while the relative abundance of Basidiomycota was lower. While the relative abundance of Ascomycota was 7.02% lower in the EAR group than in the model group, the relative abundance of Basidiomycota was 43.29% higher, indicating that EAR could effectively alleviate intestinal fungal dysfunction in SDD rats. At the genus level (Figure 5(d)), the top ten dominant fungi were *Aspergillus*, *Sebacina*, *Fusarium*, *Mortierella*, *Cladosporium*, *Acremonium*, *Penicillium*, *Filobasidium*, *Chaetomium*, and *Wallemia*, and their relative abundance is listed in Supplementary Table 4. Of these, the relative abundance of the potential pathogen, *Aspergillus*, was highest in the model group (52.08%) and was reduced to 22.08% in the EAR group. The relative abundance of the potential beneficial fungi *Mortierella*, *Filobasidium*, and *Chaetomium* increased by 1.70-, 7.16-, and 3.58-fold, respectively, in the EAR group.

### 3.7. Effect of EAR on the Intestinal Microbiome *β*-Diversity of SDD Rats

**Figure 6:**
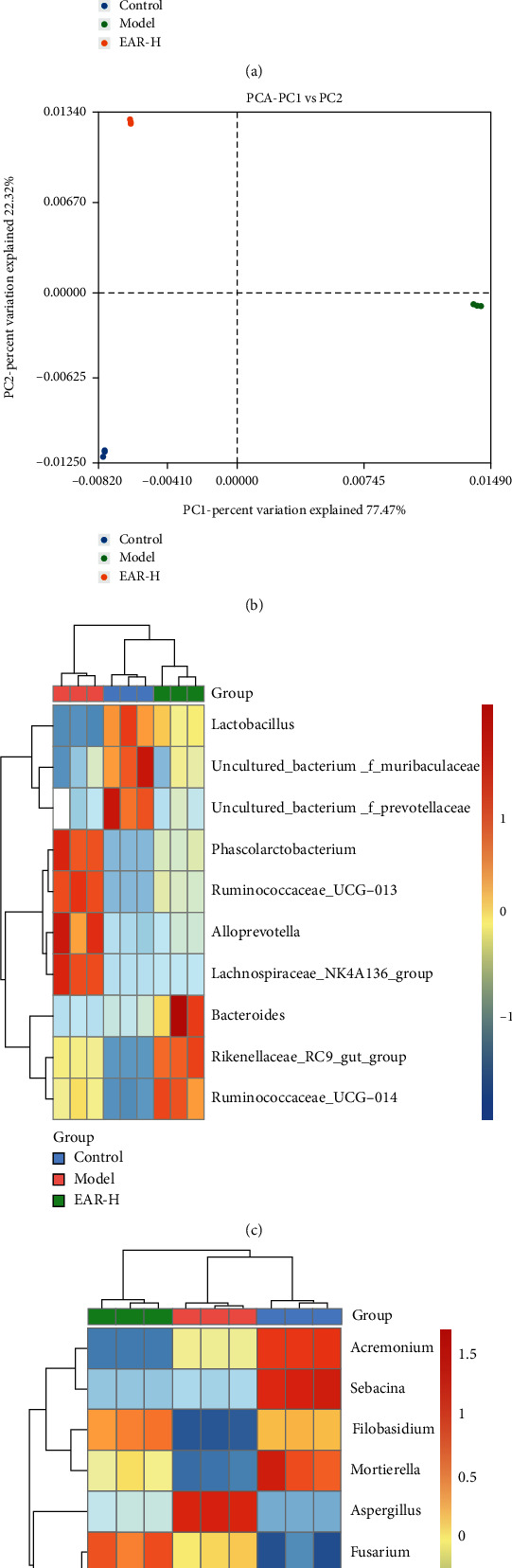
Intestinal microbiome diversity of rats in the different treatment groups. Principal component analysis (PCA) of intestinal bacterial (a) and fungal (b) communities based on OTUs; relative abundance heat map of intestinal bacterial (c) and fungal (d) communities.

A two-dimensional PCA diagram of the intestinal bacterial community diversity of rats based on OTU showed differences between the treatment groups (Figure 6(a)). Compared with the control group, the model group shifted upward along PC2, while the administration group shifted downward. The PCA diagram of the intestinal fungal community diversity (Figure 6(b)) showed that the first two main coordinates explained 77.47% and 22.32% of the total variation in the fungal community, respectively. Of these, the control and treatment groups were on the side of the coordinate axis, while the model group moved right along PC1 to the other side of the coordinate axis. Subsequently, the relative abundance thermograms of the top ten bacteria (Figure 6(c)) and fungi (Figure 6(d)) were plotted. Differences in the bacterial and fungal community diversity of each treatment group were obvious in the relative proportion values and color changes on the heat map. This analysis indicated that the diversity of the intestinal microbiome of SDD rats changed after EAR administration.

### 3.8. LEfSe Analysis of the Intestinal Microbiome of Rats in the Different Treatment Groups

**Figure 7:**
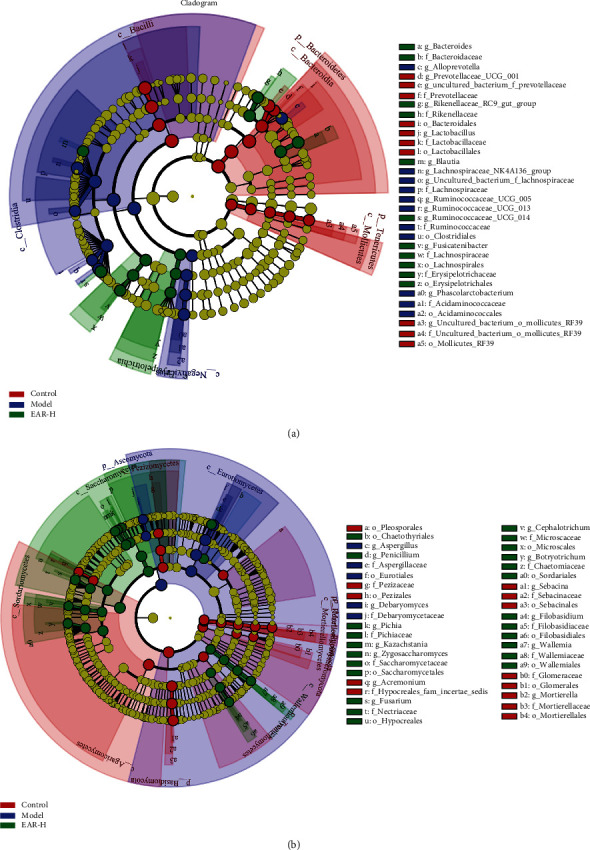
LEfSe analysis of intestinal bacteria (a) and fungi (b) of rats in the different treatment groups.

To further clarify the differences in the diversity of rat intestinal microbiota in different treatment groups, LEfSe analysis was used to explore the effects of intestinal bacteria and fungi from phylum to genus (Figure 7). Rats in the different treatment group had significantly different intestinal bacteria. A total of 40 bacterial groups with rich differences were identified, and the LDA score was >4.0 (Figure 7(a), Supplementary Figure 3). Of these, the control group had 15 (2 phyla, 3 classes, 3 orders, 3 families, and 4 genera), the model group had 13 (2 classes, 2 orders, 3 families, and 6 genera), and the EAR group had 12 (1 class, 2 orders, 4 families, and 5 genera). At the class level, Bacilli, Bacteroidia, and Mollicutes were biomarkers for the control group, Clostridia and Negativicutes were biomarkers for the model group, and Erysipelotrichia was a biomarker for the EAR group.

A total of 54 fungal groups, including 5 phyla, 8 classes, 13 orders, 14 families, and 14 genera, were identified with rich differences between rats in the different treatment groups and the LDA score was >4.0 (Figure 7(b), Supplementary Figure 4). At the class level, the most abundant fungal taxa in the control group were Agaricomycetes, Pezizomycetes, Mortierellomycetes, and Tremellomycetes, the most abundant fungal taxa in the model group was Eurotiomycetes, and the most abundant fungal taxa in the EAR group were Sordariomycetes, Saccharomycetes, and Wallemiomycetes.

## 4. Discussion

SDD syndrome is a common disease described by TCM that is characterized by changes in the physiological function of the digestive system [18]. RAR is a common herbal medicine used to treat SDD because it effectively invigorates the spleen. However, because its pharmacodynamics and mechanism of action are not well understood, the in-depth development of RAR remains limited [4]. Thus, the current study used a classical SDD model induced by rhubarb to investigate the mechanism of EAR in SDD treatment. The results showed that EAR was able to effectively control the symptoms of rhubarb-induced weight loss, appetite loss, and fecal water content. Since diarrhea is often accompanied by inflammation [19], the anti-inflammatory activity of EAR was also evaluated. EAR was shown to promote expression of the anti-inflammatory cytokine, IL-10, and inhibit accumulation of the pro-inflammatory cytokines, IL-1*β* and TNF-*α*. Among them, EAR-H was more effective than EAR-L, indicating that higher doses of EAR have better anti-inflammatory activity and can effectively maintain the balance of pro- and anti-inflammatory cytokines.

GAS, an important hormone involved in gastrointestinal digestion, affects the physiological function of the digestive system and is an objective index used to evaluate the efficacy of the “invigorating spleen” [1]. The main function of GAS is to enhance gastric antrum contraction, reduce pyloric tension, accelerate gastric emptying, accelerate gastric mucosa growth, and promote endocrine gastric acid and pepsin production [20]. In this study, EAR-H significantly increased serum GAS levels in SDD rats, indicating that this medication improved gastrointestinal function. DAO is a marker of intestinal barrier dysfunction [21] that is released into the blood when the intestinal barrier function becomes impaired. D-lactate, which is primarily formed by bacterial fermentation (e.g., Lachnospiraceae_NK4A136_group) in the intestine [22], correlates with an increase in intestinal permeability. When the intestinal mucosa is damaged and permeability increases, D-lactate can enter the blood. Thus, D-lactate in the plasma is an indirect reflection of intestinal permeability. VIP is a neurotransmitter in the central and enteric nervous systems that relaxes the intestinal smooth muscle [23] and plays an important role in the occurrence and development of various gastrointestinal diseases. DAO, D-lactate, and VIP levels were found to be higher in the serum of rats in the model group than in the control group and significantly lower in the EAR-H group than in the model group (*p* < 0.01). These findings indicated that high-dose EAR could effectively protect intestinal barrier function, alleviate intestinal mucosal injury, and relieve SDD by regulating the release of neurotransmitters. SIgA is a noninflammatory immunoglobulin that helps to maintain intestinal mucosal immunity. Its primary function is to inhibit the adhesion of bacteria, viruses, and other pathogens to prevent colonization and propagation on the surface of intestinal mucosa and limit infection [24]. The results showed that only the EAR-H group significantly promoted the accumulation of SIgA in the serum of SDD rats (*p* < 0.05), further confirming its protective effect on the intestinal mucosa, and suggesting that the ability of high-dose EAR to improve intestinal function is associated with the intestinal microbiome. Thus, in the analysis of the gut microbiome, we only studied in depth the control, model, and EAR-H groups.

The intestinal tract is a huge and complex microecosystem, with billions of microorganisms, including bacteria, fungi, and viruses [25]. The dynamic balance of intestinal microecology is crucial to the health of the host. A loss of this balance can alter the immune dynamic of the intestinal system and promote the development and progression of disease [26, 27]. The16S rDNA amplification and sequencing of intestinal bacteria showed that intestinal bacterial richness differed between the treatment groups. The model group had significantly higher richness than the control group, and after EAR treatment, the intestinal bacterial richness returned to a level comparable to the control group. A species distribution map (Figure 5) showed that Firmicutes and Bacteroidetes were the two dominant bacteria in the rat intestinal tract. These bacteria can work together to regulate host energy absorption and storage. While the main function of Firmicutes is to inhibit intestinal absorption capacity, the primary function of Bacteroidota is to promote absorption [28]. In the model group, the relative abundance of Firmicutes was increased while the relative abundance of Bacteroidota was decreased, altering energy conversion and nutrient absorption and resulting in diarrhea. EAR treatment was able to return the intestinal microbiota to normal, explaining its beneficial effect on intestinal health. At the genus level, the relative abundance of bacteria that are beneficial to intestinal health, represented by *Lactobacillus*, was lower in the model group. *Lactobacillus* is an anaerobic or facultative anaerobic gram-positive bacteria, which can protect gastric mucosa, improve intestinal function, prevent diarrhea, and promote digestion [29]. After EAR treatment, *Lactobacillus* was enriched, restoring intestinal function and reconstructing the intestinal microbiota, illustrating why it has a therapeutic effect on SDD rats. In contrast, the relative abundance of *Alloprevotella* increased in the model group and decreased following EAR treatment. *Alloprevotella* is thought to be involved in intestinal inflammation, and its increased relative abundance is an important marker of inflammation [30]. This result further indicated that SDD can cause intestinal damage that induces inflammation, while EAR can protect the intestine and inhibit the occurrence of inflammation. The intestinal bacterial diversity of rats differed significantly between the model group and the control group (Figure 6(a)), while the EAR group trended away from the model group. Importantly, it should also be noted that the intestinal bacterial diversity of the EAR group differed from the control group, indicating that EAR may adjust the intestinal bacteria and shape a new intestinal bacterial ecology. LEfSe analysis was used to further clarify differences in the diversity of intestinal bacteria in the different treatment groups (Figure 7(a)). The biomarkers in each group demonstrated the influence of EAR on the diversity of the microbiome of SDD rats.

Fungi are ubiquitous in various environments and indispensable members of the intestinal ecosystem [13]. The study of intestinal fungi is much more limited than bacteria, however, because they only account for a small proportion of human symbiotic microorganisms and most are uncultured. In recent years, the intestinal fungal biome has attracted more attention due to its potential correlation with various digestive diseases [14]. In the current study, ITS amplification and sequencing analysis of intestinal fungi showed notable differences in its richness and diversity among the different treatment groups. The model group had lower richness and diversity than the control group, but increased after EAR treatment, indicating that this medicine played an important regulatory role on the intestinal fungal biome. A species distribution map (Figure 5) showed that Ascomycota and Basidiomycota were the dominant phyla in rats. In the model group, the relative abundance of Ascomycota increased, the relative abundance of Basidiomycota decreased, and the microecology of intestinal fungi was disordered. After EAR treatment, the abundance ratio of Basidiomycota to Ascomycota gradually recovered, indicating that EAR had a positive regulatory effect on the intestinal fungi. At the genus level, the relative abundance of the potential pathogen, *Aspergillus*, was notably higher in the model group and decreased after administration. Purzycki and Shain showed that *Aspergillus* can live in the gastrointestinal tract and gradually release toxins such as glial toxin into the blood [31]. *Aspergillus* is also associated with an increase in IL-6 production in the intestinal mucosa [32]. These findings further indicate that EAR plays an anti-inflammatory role in the intestinal microenvironment. Similarly, the relative abundance of potential beneficial bacteria represented by *Filobasidium* increased after EAR administration, indicating that it is a marker of reduced inflammation [33]. Changes in the diversity of rat intestinal fungi were the same as those of bacteria. After EAR treatment, they all had positive regulatory effects, but remained different from the control group. LEfSe analysis showed the diversity of the intestinal fungi among the different treatment groups in more detail and illustrated that the most abundant fungal groups differed. This finding further supported that EAR could alleviate SDD in rats by restoring and shaping the diversity of intestinal fungi.

In summary, this study indicates that EAR has a multi-target effect on SDD remission. First, EAR regulates the balance of proinflammatory (TNF-*α*, IL-1*β*) and anti-inflammatory (IL-10) cytokines. Second, EAR has a significant regulatory and shaping effect on the intestinal flora of SDD rats. In addition, EAR improves the pathological damage of the colon by restoring cell tissue. While EAR plays a key role in improving SDD, the specific pathway of this regulation remains unclear. Future studies are needed to understand the molecular mechanism by which EAR regulates intestinal inflammatory immunity and the intestinal flora at the DNA or protein level.

## 5. Conclusions

This study demonstrated that EAR reduces fecal moisture content, enhances anti-inflammatory ability, slows gastric emptying, and improves intestinal mucosal injury, thereby promoting intestinal health. Analysis of the intestinal microbiome showed that EAR alleviated SDD by increasing the abundance of beneficial bacteria, reducing the abundance of potential pathogenic fungi to restore and shape intestinal microenvironment, and protecting the intestinal mucosa. These results indicated that EAR has a strong therapeutic effect on SDD and may serve as a promising drug for SDD treatment.

## Figures and Tables

**Table 1:** Component analysis of ethanolic extract of rice water fried Atractylodis Rhizoma.

Number	RT-time	Compound	Content (%)
1	12.09	Carvacrol	0.49
2	12.34	3-Methyl-4-isopropylphenol	0.14
3	15.87	Hinesol	0.44
4	16.00	*β*-Eudesmol	0.83
5	16.11	*α*-Bisabolol	0.17
6	16.28	2-Nitrodiphenyl	18.67
7	16.86	4-(2-Phenylethenyl)pyridazine	11.92
8	17.60	5,8,11,14-Icosatetrynoic acid	19.70
9	17.80	Methyl palmitate	1.73
10	19.11	7,10-Octadecadienoic acid methyl ester	7.40

## Data Availability

The data used to support the findings of this study are included within the article and the supplementary information files.

## Supplementary Materials

Supplementary Materials Supplementary 1. Supplementary Figure 1: GC-MS chromatogram of EAR. Supplementary 2. Supplementary Figure 2: intestinal bacteria (A) and fungi (B) OTU Venn diagram. Supplementary 3. Supplementary Figure 3: LDA score of significantly different bacteria in each group. Supplementary 4. Supplementary Figure 4: LDA score of significantly different fungi in each group. Supplementary 5. Supplementary Table 1: relative abundance (%) of bacterial phyla in all treatments. Supplementary 6. Supplementary Table 2: relative abundance (%) of bacterial genera in all the treatments. Supplementary 7. Supplementary Table 3: relative abundance (%) of fungal phyla in all treatments. Supplementary 8. Supplementary Table 4: relative abundance (%) of fungal genera in all the treatments.
