# Supplementary material for: Rice Water-Fried Atractylodis Rhizoma Relieves Spleen Deficiency Diarrhea by Regulating the Intestinal Microbiome
Source: Oxid Med Cell Longev. 2023 Feb 7;2023:1983616. doi: 10.1155/2023/1983616 (PMC9928513; doi:10.1155/2023/1983616)
Supplement: Supplementary Materials — Supplementary 1. Supplementary Figure 1: GC-MS chromatogram of EAR. Supplementary 2. Supplementary Figure 2: intestinal bacteria (A) and fungi (B) OTU Venn diagram. Supplementary 3. Supplementary Figure 3: LDA score of significantly different bacteria in each group. Supplementary 4. Supplementary Figure 4: LDA score of significantly different fungi in each group. Supplementary 5. Supplementary Table 1: relative abundance (%) of bacterial phyla in all treatments. Supplementary 6. Supplementary Table 2: relative abundance (%) of bacterial genera in all the treatments. Supplementary 7. Supplementary Table 3: relative abundance (%) of fungal phyla in all treatments. Supplementary 8. Supplementary Table 4: relative abundance (%) of fungal genera in all the treatments. [file 1983616.f1.docx]

Supplementary Material

# 1 Supplementary Figures and Tables

## 1.1 Supplementary Figures


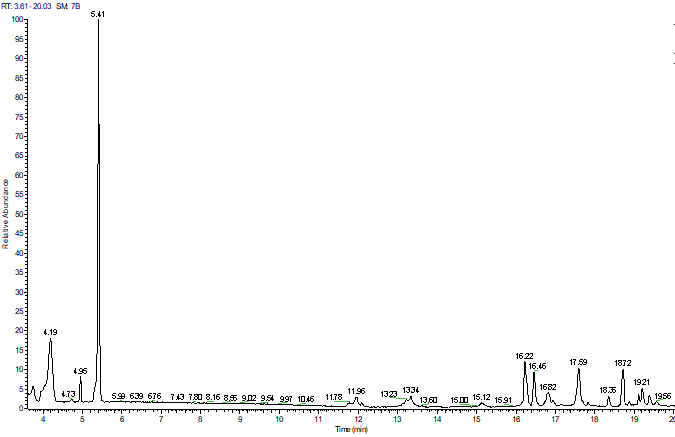


**Supplementary Figure 1.** GC-MS chromatogram of EAR.

**
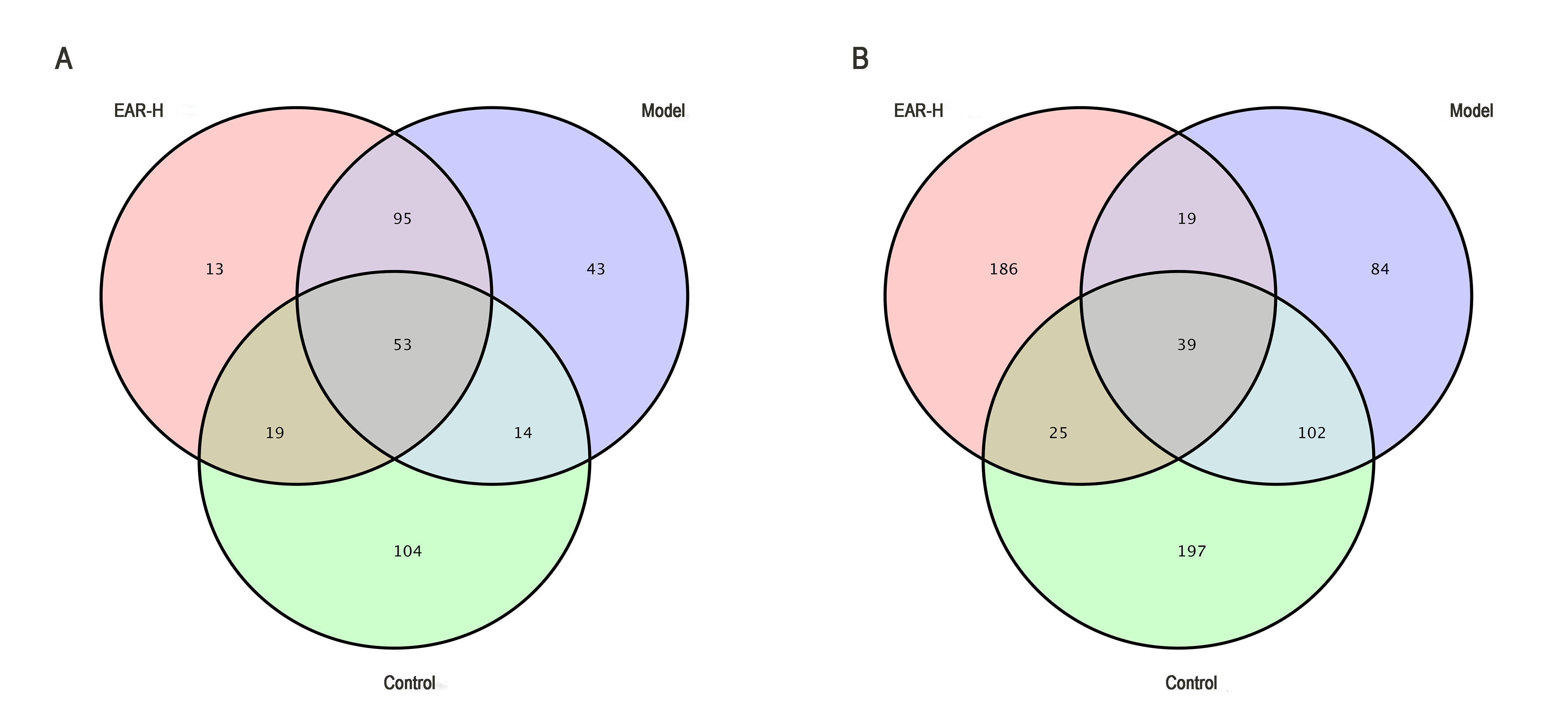
**

**Supplementary Figure 2.** Intestinal bacteria (A) and fungi(B) OTUs Venn diagram.

**
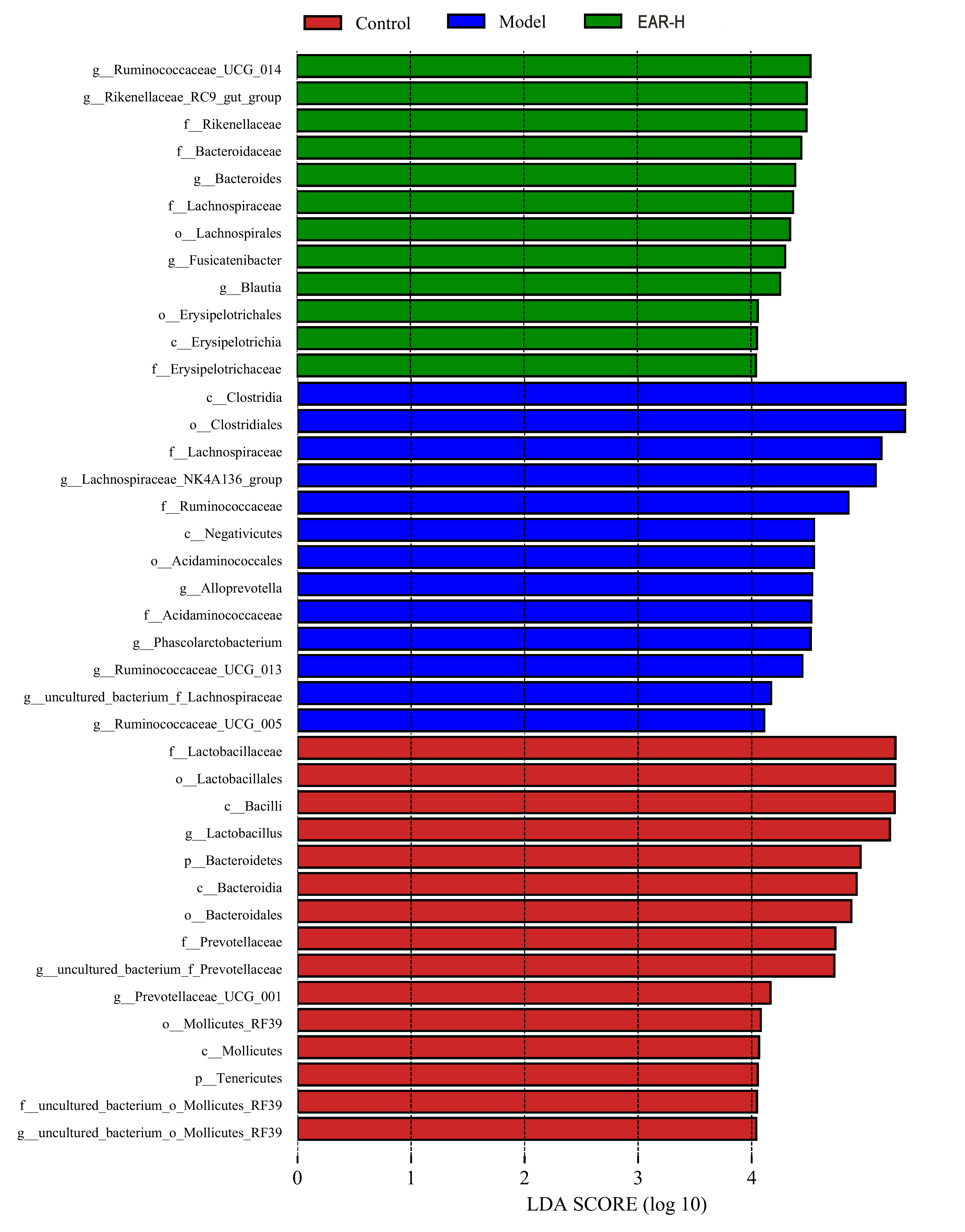
**

**Supplementary Figure 3.** LDA score of significantly different bacteria in each group. Taxa with LDA values > 4.

**
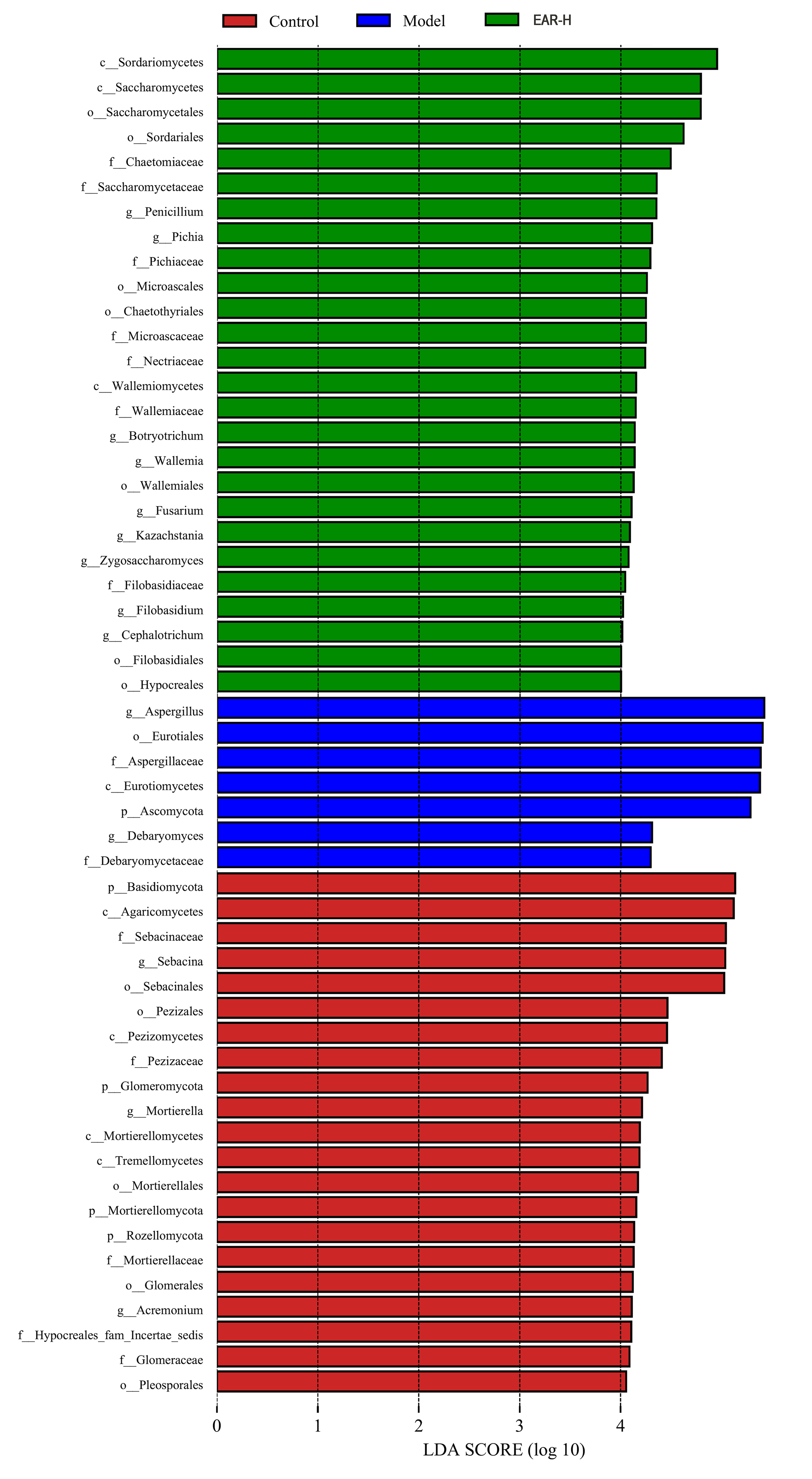
**

**Supplementary Figure 4.** LDA score of significantly different fungi in each group. Taxa with LDA values > 4.

**1.2 Supplementary Tables**

Supplementary Table 1

| **Table S1.** Relative abundance ( % ) of bacterial phyla in all treatments. | | | |
| --- | --- | --- | --- |
| phylum | Control | Model | EAR-H |
| Firmicutes | 47.92 | 62.64 | 58.96 |
| Bacteroidetes | 46.33 | 28.71 | 37.51 |
| Verrucomicrobiota | 0.03 | 6.64 | 0.06 |
| Tenericutes | 2.93 | 1.08 | 0.82 |
| Proteobacteria | 1.01 | 0.46 | 2.26 |
| Actinobacteria | 0.89 | 0.08 | 0.32 |
| Patescibacteria | 0.83 | 0.25 | 0.06 |
| Desulfobacterota | 0.00 | 0.09 | 0.00 |
| Planctomycetes | 0.05 | 0.00 | 0.00 |
| Cyanobacteria | 0.00 | 0.05 | 0.00 |

Note: (a) Values are mean (n = 3).

Supplementary Table 2

| **Table S2.** Relative abundance (%) of bacterial genera in all the treatments. | | | |
| --- | --- | --- | --- |
| genus | Control | Model | EAR-H |
| *Lactobacillus* | 41.90 | 7.01 | 28.57 |
| *uncultured_bacterium_f_Muribaculaceae* | 26.17 | 14.72 | 17.49 |
| *Lachnospiraceae_NK4A136_group* | 0.15 | 23.80 | 0.29 |
| *uncultured_bacterium_f_Prevotellaceae* | 12.73 | 1.45 | 2.54 |
| *Ruminococcaceae_UCG-014* | 0.29 | 4.01 | 6.85 |
| *Alloprevotella* | 1.04 | 8.01 | 1.80 |
| *Rikenellaceae_RC9_gut_group* | 0.00 | 3.15 | 6.36 |
| *Phascolarctobacterium* | 0.00 | 6.93 | 1.67 |
| *Bacteroides* | 1.00 | 0.68 | 5.95 |
| *Ruminococcaceae_UCG-013* | 0.00 | 5.85 | 1.42 |

Note: (a) Values are mean (n = 3).

Supplementary Table 3

| **Table S3.** Relative abundance ( % ) of fungal phyla in all treatments. | | | |
| --- | --- | --- | --- |
| phylum | Control | Model | EAR-H |
| Ascomycota | 48.17 | 84.56 | 78.63 |
| Basidiomycota | 36.34 | 8.89 | 12.74 |
| Mortierellomycota | 4.97 | 2.14 | 3.63 |
| Rozellomycota | 4.98 | 3.03 | 2.04 |
| Glomeromycota | 4.47 | 0.77 | 1.00 |
| Chytridiomycota | 0.65 | 0.31 | 0.70 |
| Blastocladiomycota | 0.04 | 0.00 | 0.00 |
| Unclassified | 0.38 | 0.29 | 1.26 |

Note: (a) Values are mean (n = 3).

Supplementary Table 4

| **Table S4.** Relative abundance (%) of fungal genera in all the treatments. | | | |
| --- | --- | --- | --- |
| genus | Control | Model | EAR-H |
| *Aspergillus* | 3.62 | 52.08 | 11.50 |
| *Sebacina* | 22.08 | 0.79 | 0.00 |
| *Fusarium* | 2.39 | 4.32 | 5.07 |
| *Mortierella* | 4.97 | 2.14 | 3.63 |
| *Cladosporium* | 1.79 | 1.86 | 4.09 |
| *Acremonium* | 4.22 | 1.98 | 0.10 |
| *Penicillium* | 0.73 | 0.20 | 4.63 |
| *Filobasidium* | 2.27 | 0.35 | 2.48 |
| *Chaetomium* | 1.49 | 0.73 | 2.63 |
| *Wallemia* | 0.12 | 1.76 | 2.79 |

Note: (a) Values are mean (n = 3).
